# The three muscle layers in the pyloric sphincter and their possible function during antropyloroduodenal motility

**DOI:** 10.1038/s41598-021-99463-x

**Published:** 2021-10-11

**Authors:** Mi-Sun Hur, Seunggyu Lee, Tong Mook Kang, Chang-Seok Oh

**Affiliations:** 1 Department of Anatomy, Catholic Kwandong University College of Medicine, Gangneung, Korea; 2 Division of Applied Mathematical Sciences, Korea University, Sejong, Korea; 3 Biomedical Mathematics Group, Pioneer Research Center for Mathematical and Computational Sciences, Institute for Basic Science, Daejeon, 34126 Korea; 4 Department of Physiology, Sungkyunkwan University School of Medicine, Suwon, Korea; 5 Department of Anatomy and Cell Biology, Sungkyunkwan University School of Medicine, 2066, Seobu-ro, Jangan-gu, Suwon-si, 16419 Gyeonggi-do Korea

**Keywords:** Anatomy, Medical research

## Abstract

This study was conducted to determine the muscular arrangement of the human pyloric sphincter using a comprehensive approach that involved microdissection, histology, and microcomputed tomography (micro‐CT). The stomachs of 80 embalmed Korean adult cadavers were obtained. In all specimens, loose muscular tissue of the innermost aspect of the sphincter wall ran aborally, forming the newly found inner longitudinal muscle bundles, entered the duodenum, and connected with the nearby circular bundles. In all specimens, approximately one-third of the outer longitudinal layer of the sphincter entered its inner circular layer, divided the circular layer into several parts, and finally connected with the circular bundles. Anatomical findings around the sphincter were confirmed in micro-CT images. The sphincter wall comprised three layers: an inner layer of longitudinal bundles, a middle layer of major circular and minor longitudinal bundles, and an outer layer of longitudinal bundles. The stomach outer longitudinal bundles were connected to the sphincter circular bundles. The inner longitudinal bundles of the sphincter were connected to the adjacent circular bundles of the duodenum.

## Introduction

The pyloric sphincter does not appear to serve the simple role of a traditional sphincter given that its muscular anatomy, physiology, and pharmacology are clearly different from those in adjacent regions^1^. The sphincter originates from a mixture of the gastric and duodenal regions, which may be related to its permanent form and function^2^. Pylorus smooth muscle also behaves differently to muscles in adjacent regions^1^. The innervation density in the pylorus is also higher than those in the duodenum and antrum of guinea pigs^3^.

The anatomical arrangement of the human pyloric sphincter musculature has not been reported on in detail. Layers other than inner circular and outer longitudinal layers of the sphincter have also not been investigated. Routine dissections in the authors’ institution have frequently revealed an additional layer inside the innermost part of the sphincter layer. There have been a few descriptions of the longitudinal muscle bundles of the sphincter outer layer that run inward to the sphincter. Goss (1973)^4^ suggested that the sphincter contained some longitudinal bundles of the pyloric canal outer layer that ran inward to interlace with the ring’s circular bundles. Hollinshead (1982)^5^ depicted that the longitudinal muscles of the stomach were interrupted at the pyloric end of the stomach, rather than continuing alongside the longitudinal muscles of the duodenum. Torgersen (1942)^2^ reported that within the pyloric canal, radiation inward to the sphincter of the longitudinal musculature occurred to some degree, which was prominent in horses, dogs, and humans. However, the ends of the longitudinal bundles entering the sphincter have not been investigated previously.

This study was conducted to determine the muscular arrangement of the sphincter, focusing on its longitudinal bundles and their relationship with the circular bundles of the sphincter and duodenum using a comprehensive approach involving microdissection, histology, microcomputed tomography (micro‐CT), and numerical simulations.

## Results

### The longitudinal muscle bundles entering the pyloric sphincter

**Figure 1:**
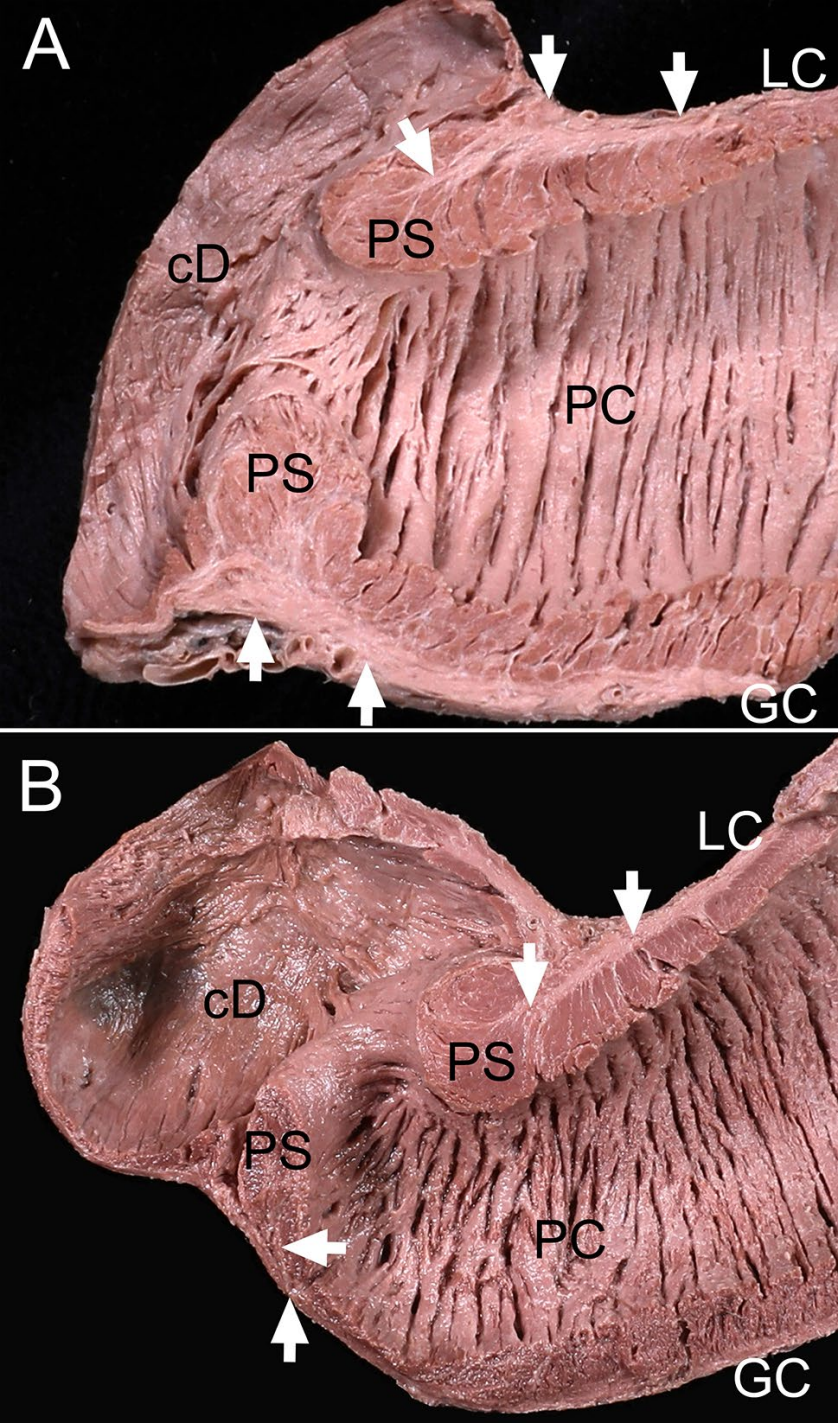
The thickest longitudinal muscle bundles at the pyloric sphincter (PS) wall and the longitudinal bundles entering the PS. (**A**) The longitudinal bundles (arrows) of the stomach became thicker at the pyloric canal (PC) wall, especially on the side of the lesser curvature (LC), and the longitudinal bundles (arrows) were the thickest at the PS wall in a longitudinal section through the PS wall including the LC and greater curvature (GC). (**B**) Some longitudinal bundles (arrows) entering the PS in a longitudinal section of the PS were cut including the LC and GC. The longitudinal bundles were more prominent in the PS on the LC side than the GC side. *cD* circular bundles of the duodenum.

**Figure 2:**
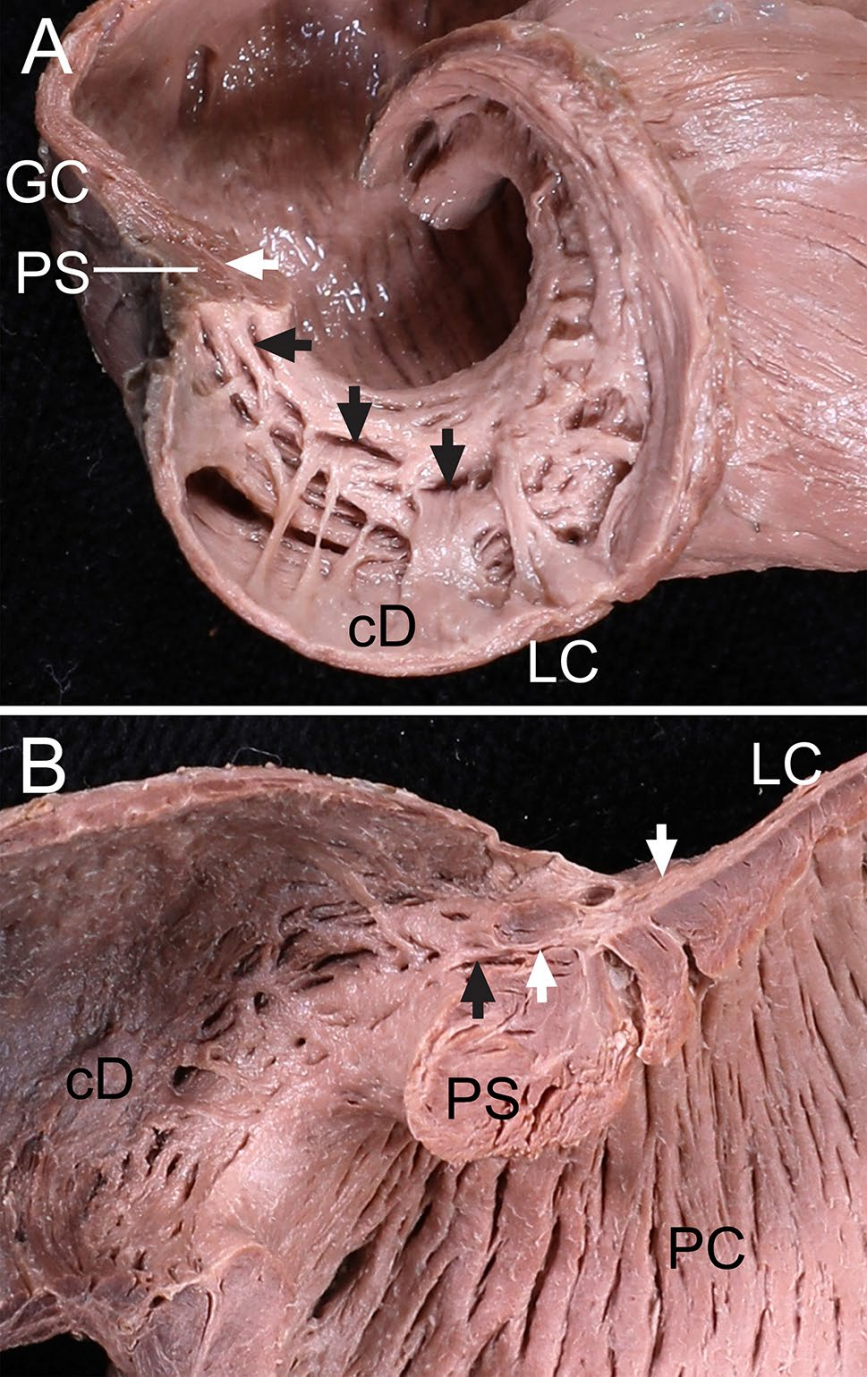
The pyloric sphincter (PS) divided according to the direction of the longitudinal bundles entering it. (**A**) Some longitudinal bundles (white arrow) divided the PS into several parts in a longitudinal section including the greater curvature (GC). The divided parts (black arrows) of the PS were not separated completely, but were connected to each other. (**B**) Some longitudinal bundles (white arrows) entering the PS were connected to the aboral circular bundles (black arrow) of the PS in a longitudinal section including the lesser curvature (LC) and GC. *cD* circular bundles of the duodenum.

The layer of longitudinal muscle bundles in the pyloric canal became thicker as it approached the sphincter, especially along the lesser curvature that occupied the thickest portion at the sphincter wall and became thinner as it exited the sphincter. In all specimens, approximately one-third of the outer longitudinal layer of the sphincter entered its inner circular layer (Fig. 1). These bundles divided the circular layer into several parts, and ended when it connected with the sphincter circular bundles (Fig. 2). The parts divided by the longitudinal bundles were not separated completely, but were connected to each other.

### The inner longitudinal muscle bundles of the sphincter

Loose muscular tissue was found at the innermost aspect of the sphincter wall. These loose muscular bundles had an irregular arrangement.

**Figure 3:**
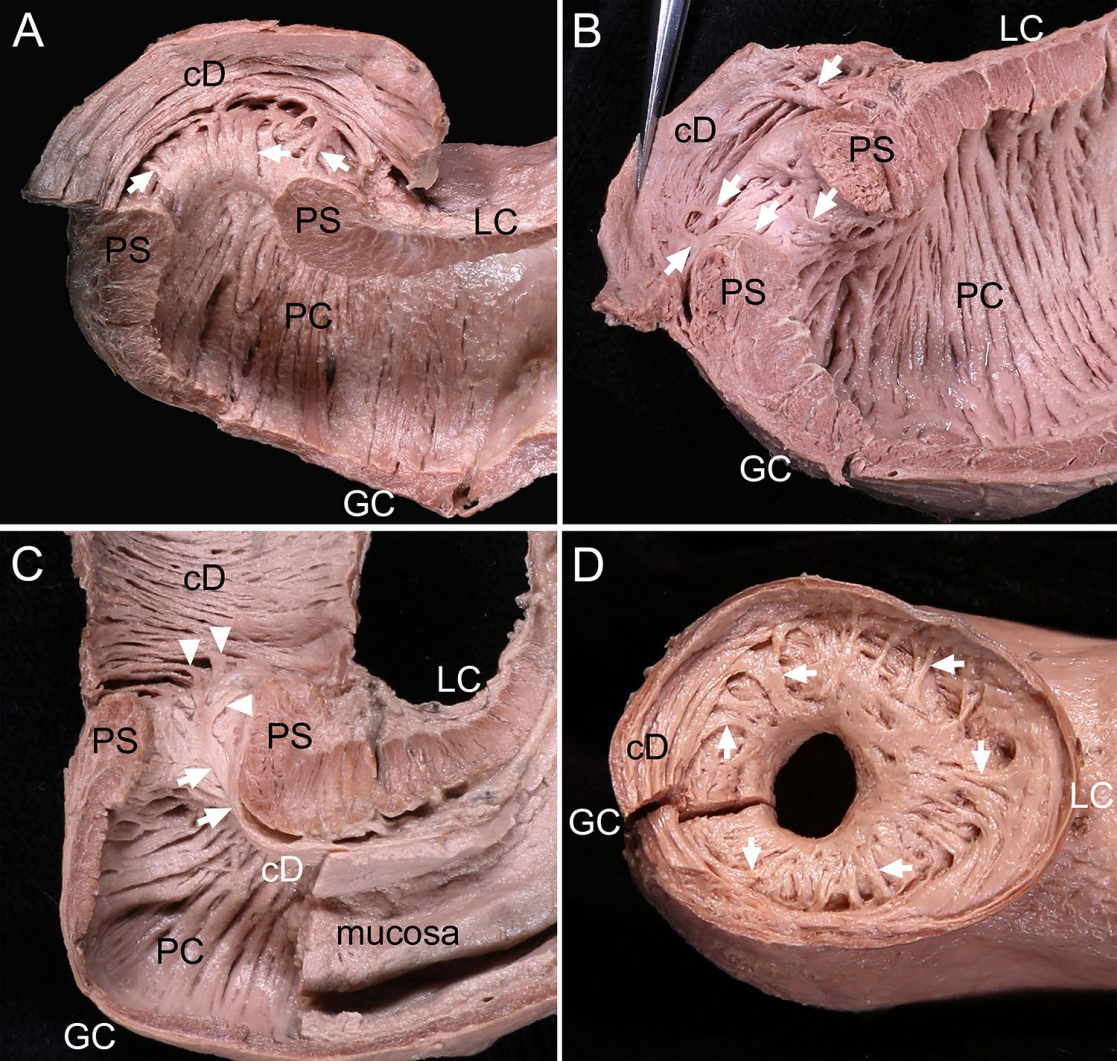
The inner longitudinal bundles on the internal surface of the pyloric sphincter (PS) wall in a longitudinal section including the lesser curvature (LC) and greater curvature (GC). (**A**) The inner longitudinal bundles (arrows) were divided from the muscle tissue of the internal surface of the PS wall and were connected to the adjacent circular bundles of the duodenum (cD). (**B**) The inner longitudinal bundles (arrows) were divided from the irregularly arranged muscle tissue at the PS internal surface. (**C**) The muscularis mucosae (arrows) was attached to the internal surface of the PS wall, and was divided into several muscle bundles (arrowheads) connecting to the adjacent cD. (**D**) The inner longitudinal bundles (arrows) were connected to the adjacent cD in irregular and radial directions in the duodenal aspect of the pyloric orifice. *PC* pyloric canal.

In all specimens, the loose muscular tissue ran aborally to form the inner longitudinal bundles, entered the duodenum, and then connected with the nearby circular bundles (Fig. 3). Part of the muscularis mucosae on a specimen’s stomach was observed as the muscular tissue of the internal surface of the sphincter wall. The inner longitudinal bundles were mostly present at the aboral end of the sphincter, and their number, length, and thickness varied between specimens.

### Sphincter muscle bundles arrangement in relation to the duodenum and pyloric canal

**Figure 4:**
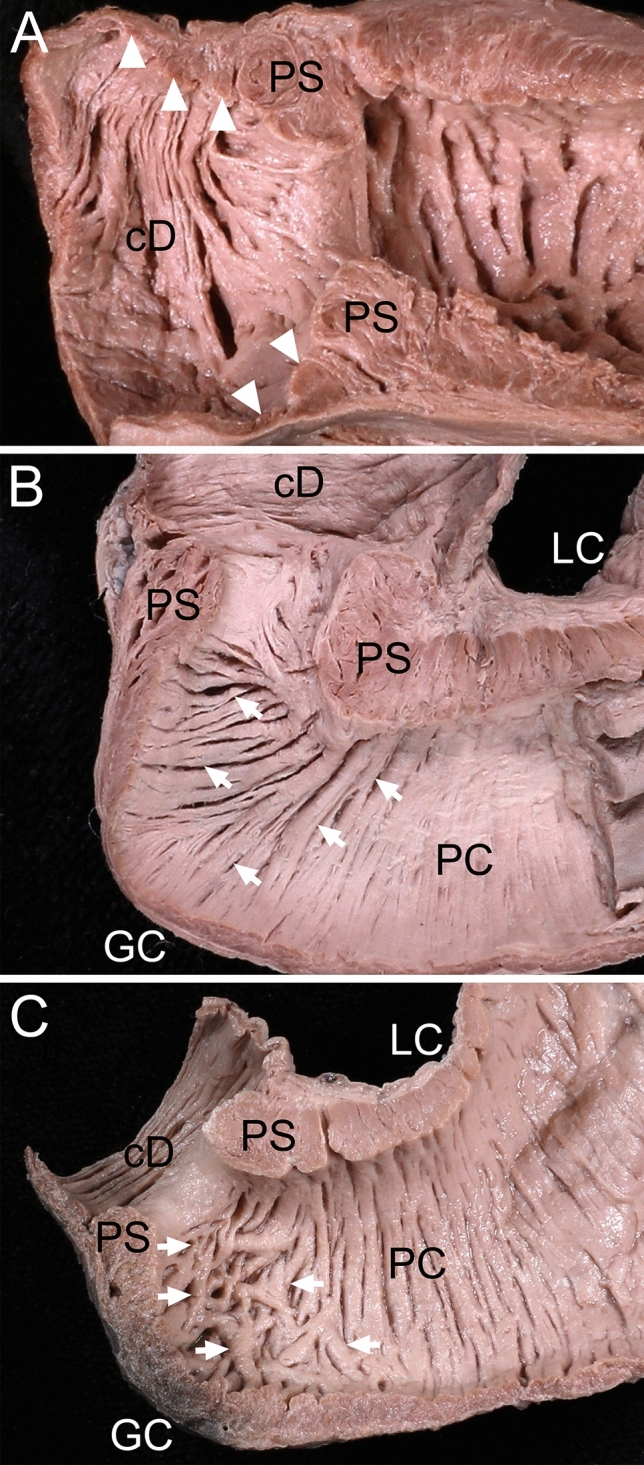
Arrangement of muscle bundles between the pyloric sphincter (PS), duodenum, and the pyloric canal (PC). (**A**) The circular bundles of the duodenum (cD) (arrowheads) adjacent to the PS were the thickest, and gradually reduced in thickness between the PS and duodenum in a longitudinal section between the lesser curvature (LC) and greater curvature (GC). (**B**) The circular bundles (arrows) of the PS on the LC side spread out to connect to the circular bundles of the aboral end of the PC on the GC side. (**C**) The circular bundles (arrows) of the aboral end of the PC adjacent to the PS were irregularly arranged. The PS was cut along a longitudinal section through the PS wall including the LC and GC.

The aboral end of the pyloric canal circular bundles converged on the sphincter at the lesser curvature side, which made the sphincter thicker here than at the greater curvature in longitudinal sections including the lesser and greater curvatures. The circular layer of the duodenum was thicker adjacent to the sphincter along the greater curvature than along the lesser curvature in all specimens (Fig. 4).

### Histological features of the sphincter

**Figure 5:**
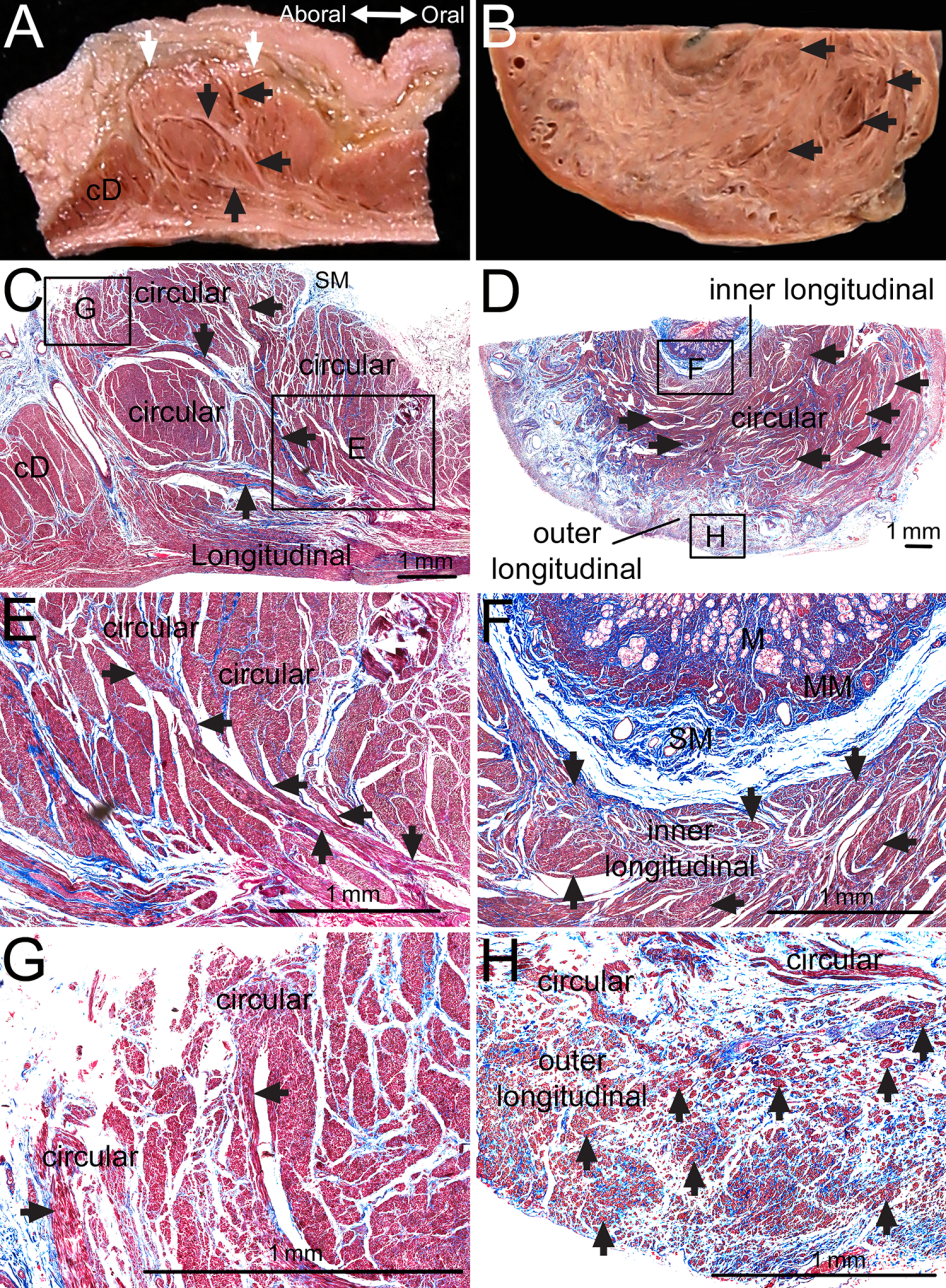
Histological examinations of the pyloric sphincter (PS) with the corresponding macroscopic observations. (**A**,**C**) In a longitudinal section of the PS (white arrows) on the lesser curvature (LC) side, some longitudinal bundles (black arrows) entered the PS and divided it into several parts, as seen in macroscopic observations (**A**) and the corresponding histological examinations (**C**). (**C**) The internal parts of the PS were divided more than the external parts by the muscle fascia and connective tissue connecting to the submucosae (SM). (**E**,**G**) Are enlarged views of the rectangular insets in (**C**). (**E**) Some longitudinal bundles (black arrows) that entered the PS were divided into several bundles that intermingled with and connected to the sphincteric circular bundles. (**G**) Some longitudinal bundles (black arrows) at the aboral end of the PS that entered the PS were connected to the circular bundles of the PS. (**B**,**D**) There were three muscle layers in an axial section including the lesser and greater curvatures of the aboral end of the PS (inner longitudinal; arrows, thick circular, and outer longitudinal muscle layers) seen in macroscopic observations (**B**) and the corresponding histological examinations (**D**). (**F**,**H**) Are enlarged views of the rectangular insets in (**D**). Some inner longitudinal bundles were found between the thick circular bundles of the PS. (**F**) The inner longitudinal bundles (arrows) were arranged adjacent to the SM in the aboral end of the PS. (**H**) Outer longitudinal bundles (arrows) were located external to the circular bundles in the PS wall. *M* mucosae, *MM* muscularis mucosae.

Histological observations indicated the general arrangement of the longitudinal and circular bundles within the sphincter and the insertion of the longitudinal bundles entering the sphincter inner circular layer, which divided into several bundles that intermingled and connected with the sphincteric circular bundles. The muscle fascia and connective tissue arising from the submucosae divided the internal portion of the circular layer into more parts than its external portion. Three muscle layers were observed on the axial section including the lesser and greater curvatures of the aboral end of the sphincter: the inner longitudinal muscle layer; the middle thick circular muscle layer, which was intervened by some longitudinal bundles; and outer longitudinal muscle layer. The inner longitudinal bundles were usually found adjacent to the submucosae at the aboral end of the sphincter on serial axial sections including the lesser and greater curvatures from the sphincter to the duodenum (Fig. 5).

### Analysis of the sphincter using micro‐CT images

**Figure 6:**
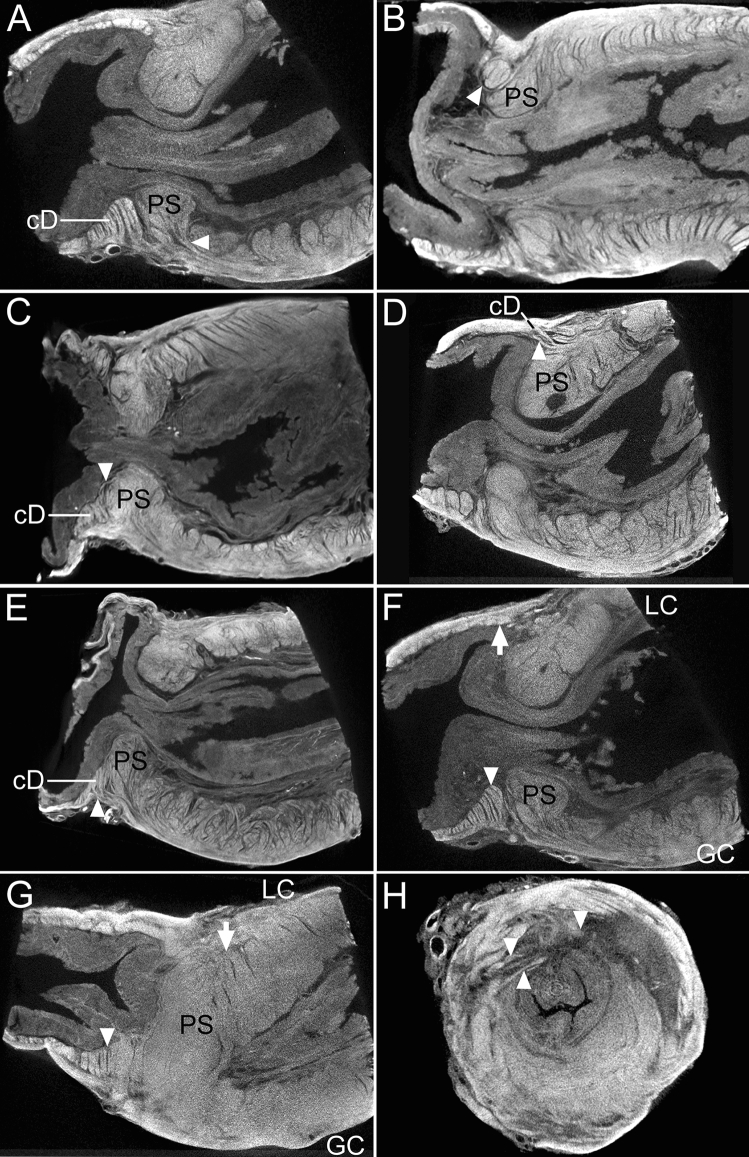
Microcomputed tomography images of the pyloric sphincter (PS). (**A**) Some outer longitudinal fibers (arrowhead) entered the PS in the longitudinal plane. (**B**) Some longitudinal fibers (arrowhead) entering the PS were connected to the aboral circular PS bundles in the longitudinal plane. (**C**) Some inner longitudinal bundles (arrowhead) were divided at the internal surface of the intermediate PS wall to connect to the circular bundles of the duodenum (cD) (arrowhead), adjacent to the PS in the longitudinal plane. (**D**) Some inner longitudinal bundles (arrowhead) that divided at the internal surface of the aboral end of the PS wall were connected to the cD adjacent to the PS in the longitudinal plane. (**E**) Some outer longitudinal bundles (arrowhead) were connected to the cD adjacent to the PS. (**F**) The cD (arrowhead) adjacent to the PS on the greater curvature (GC) side were thicker than those (arrow) on the lesser curvature (LC) side in the longitudinal plane. (**G**) In the longitudinal plane, the thick PS layer (arrow) on the LC side, and the thick duodenum muscular layer (arrowhead) on the GC side were successively arranged, crossing the gastroduodenal junction in the longitudinal plane. (**H**) In the axial plane, some muscles bundles (arrowheads) between the PS and duodenum were arranged irregularly.

Serial micro-CT scans revealed the internal arrangement of the sphincter bundles (Fig. 6), which supported the anatomical findings. The outer longitudinal bundles of the sphincter were also connected to the circular bundles of the duodenum in one specimen.

The sphincter was thicker on the lesser curvature than on the greater curvature in the longitudinal plane, while the circular bundle layer of the duodenum was thicker near the greater curvature. The thick sphincter on the lesser curvature side and the thick circular bundle layer of the duodenum on the greater curvature side were therefore successively observed, and crossed the gastroduodenal junction.

### Numerical simulations of sphincter contraction

**Figure 7:**
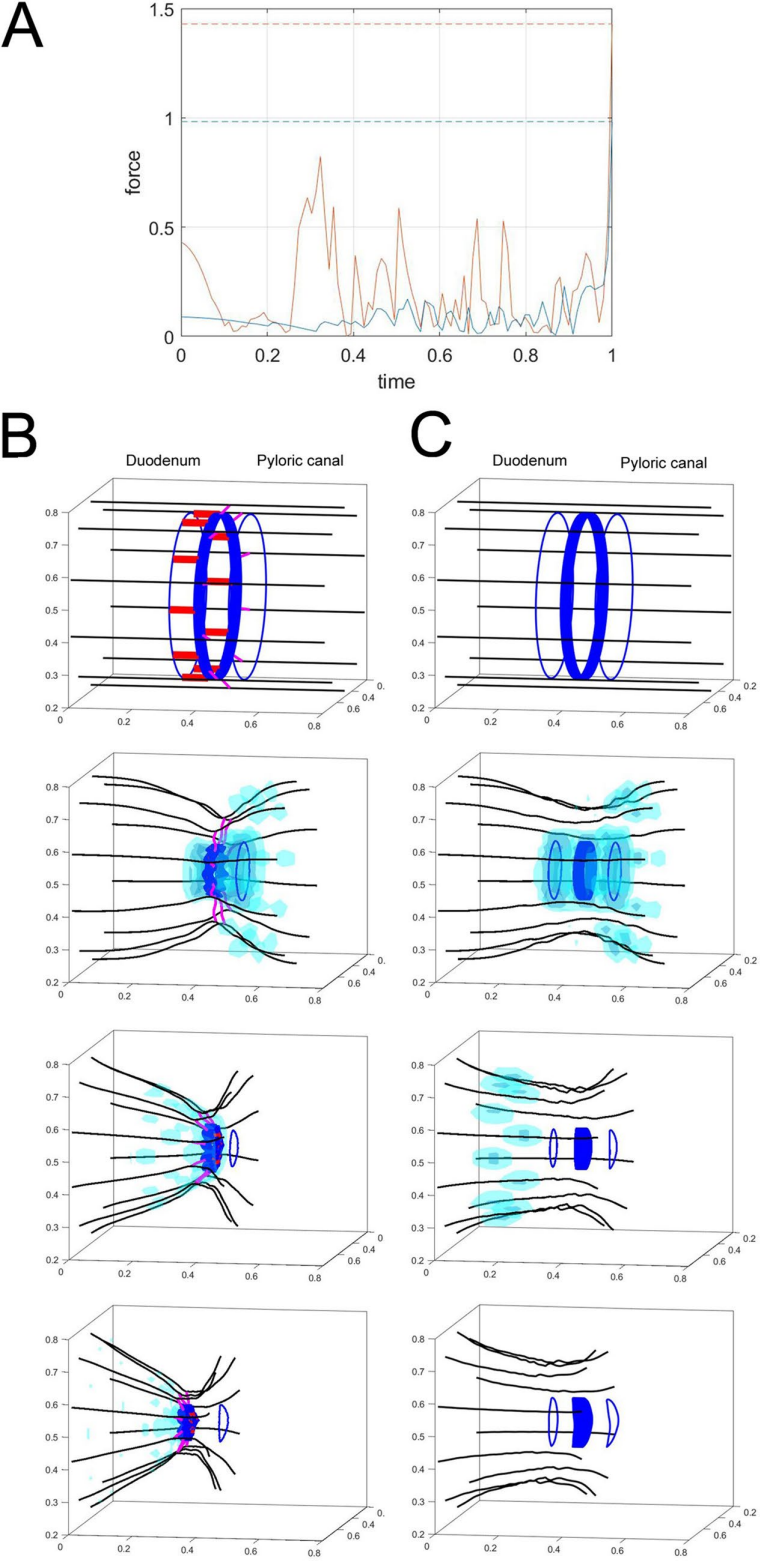
Mathematical simulation of pyloric sphincter (PS) bundle contraction. (**A**) The graph displays the mechanical simulation of the force evolution versus time for single (blue) and three (orange) longitudinal muscle layers during PS shrinking. The time and force scales were dimensionless and a Weber number is 8 in the simulations. In the early stage, the force generated by the three longitudinal muscle layers was approximately fourfold greater than that of the single layered muscle. The meaningful difference between the forces from the single and three muscle layers was evident during one complete shrinking process. The maximum force driven by the three muscle layers as approximately 1.5-fold greater than that in the single layered muscle in the final stage. (**B**,**C**) An evolution of force distribution (green) generated by the bundles is evident when the outer layer of longitudinal bundles (black) and circular bundles (blue) contract with (**B**) and without (**C**) the inner layer of longitudinal bundles (red) and the middle layer of longitudinal bundles (pink). The forces are similarly distributed in both (**B**) and (**C**) at the initial state. The distribution of the generated force becomes more concentrated on the PS (thicker blue) over time. Note that the distribution is more focused on the PS and the PS shrinks more alongside the inner and middle layers of longitudinal bundles (**B**). In the final stage, a greater contraction force is applied to the PS and oral duodenum regions and prevents regurgitation for longer periods when the inner and middle layers of longitudinal bundles were present (**B**).

The sphincter contraction was simulated using computational and mathematical models, and the forces generated by the three longitudinal bundles (outer, middle, and inner longitudinal layers) were compared with the current knowledge on forces produced by a single longitudinal bundle (Fig. 7) (Supplementary information 1). During the early stages of the simulation, the contractile force generated by the three layers was approximately fourfold greater than that of a single layer. This increased force was maintained throughout the simulation process. The maximum force of the three layers was approximately 1.5-fold greater than the single layer contraction in the simulation’s final stage. The simulation model in this study did not include the connection between the longitudinal and circular bundles, which would demonstrate better the contribution of longitudinal bundles to the function of sphincter.

## Discussion

**Figure 8:**
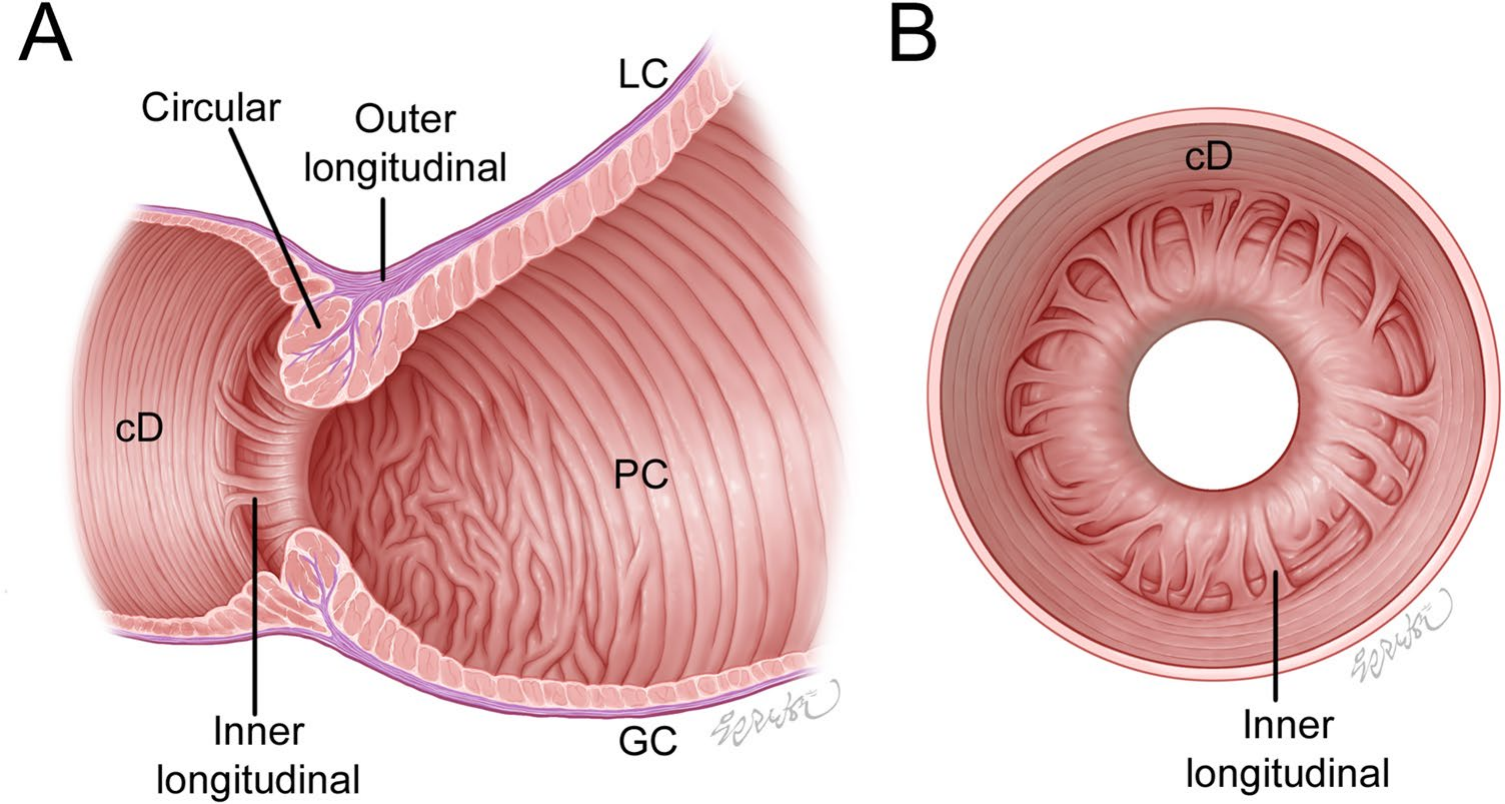
Schematic of the pyloric sphincter (PS) general pattern in a longitudinal section including the lesser curvature (LC) and greater curvature (GC). (**A**) and the duodenal aspect of the pyloric orifice (**B**). (**A**) The wall of the PS comprised three layers: outer longitudinal bundles, intermediate main circular and minor longitudinal bundles originating from the stomach, and inner longitudinal bundles connecting the PS to the duodenum. The longitudinal bundles that entered the PS were connected to the circular bundles of the PS. The inner longitudinal bundles were connected to the circular bundles of the duodenum (cD). The circular fibers of the PS on the LC side spread out to connect with the circular bundles of the aboral end of the pyloric canal (PC) on the GC side. The circular bundles in the aboral end of the PC adjacent to the PS were irregularly arranged. (**B**) The inner longitudinal bundles usually divided from the internal surface of the PS wall to connect to the adjacent cD in irregular and radial directions in the duodenal aspect of the pyloric orifice.

This study found that the sphincter is a complicated structure with three connected muscle layers. Hur (2020)^6^ found a large number of muscle bundles crossing and connecting the oblique and circular or longitudinal bundles on the left side of the gastric cardia and the angle of the His may increase the thickness of the muscular wall and the pressure in these areas. The presence of the newly found sphincter inner longitudinal bundles and the muscle layer connections may therefore play an important role in powerful and efficient antropyloroduodenal motility, and the following characteristics of the sphincter and its adjacent regions were indicated (Fig. 8 and Table 1):

1. The wall of the sphincter comprised three layers: an inner layer of longitudinal bundles, a middle layer of major circular and minor longitudinal bundles, and an outer layer of longitudinal bundles.
2. The outer longitudinal bundles of the stomach that entered the circular layer of the sphincter were connected to its circular bundles. The inner longitudinal bundles of the sphincter were also connected to the circular bundles of the duodenum, adjacent to the sphincter.

**Table 1:**
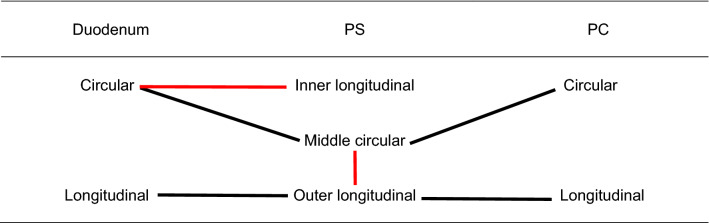
Changes and connections of the muscle layers between the pyloric canal (PC) and duodenum.

The PC has two muscle layers: the inner circular and outer longitudinal muscle bundles. The pyloric sphincter (PS) has three muscle layers: inner longitudinal, middle circular, and outer longitudinal bundles. The duodenum has two muscle layers: inner circular and outer longitudinal bundles. Some of the outer longitudinal bundles of the PS were connected to the middle circular bundles of the PS. The inner longitudinal bundles of the PS were connected to the circular bundles of the duodenum. Red lines indicate connections between the longitudinal and circular bundles. Black lines indicate continuous longitudinal or circular fibers between the PC and the PS and between the PS and duodenum.

In guinea pigs, bundles of inner longitudinal muscle running close to the antrum submucosae form a loop and contribute to the circular musculature of the pylorus^3^. The presence of inner longitudinal bundles and their connection to the circular bundles in humans and guinea pigs may indicate their importance in antropyloroduodenal motility. Simultaneous contractions of the inner and outer longitudinal bundles may improve the balance between the outer and inner walls of the sphincter during local longitudinal shortening, enhancing motility of the sphincter, and tight closing of the lumen.

**Figure 9:**
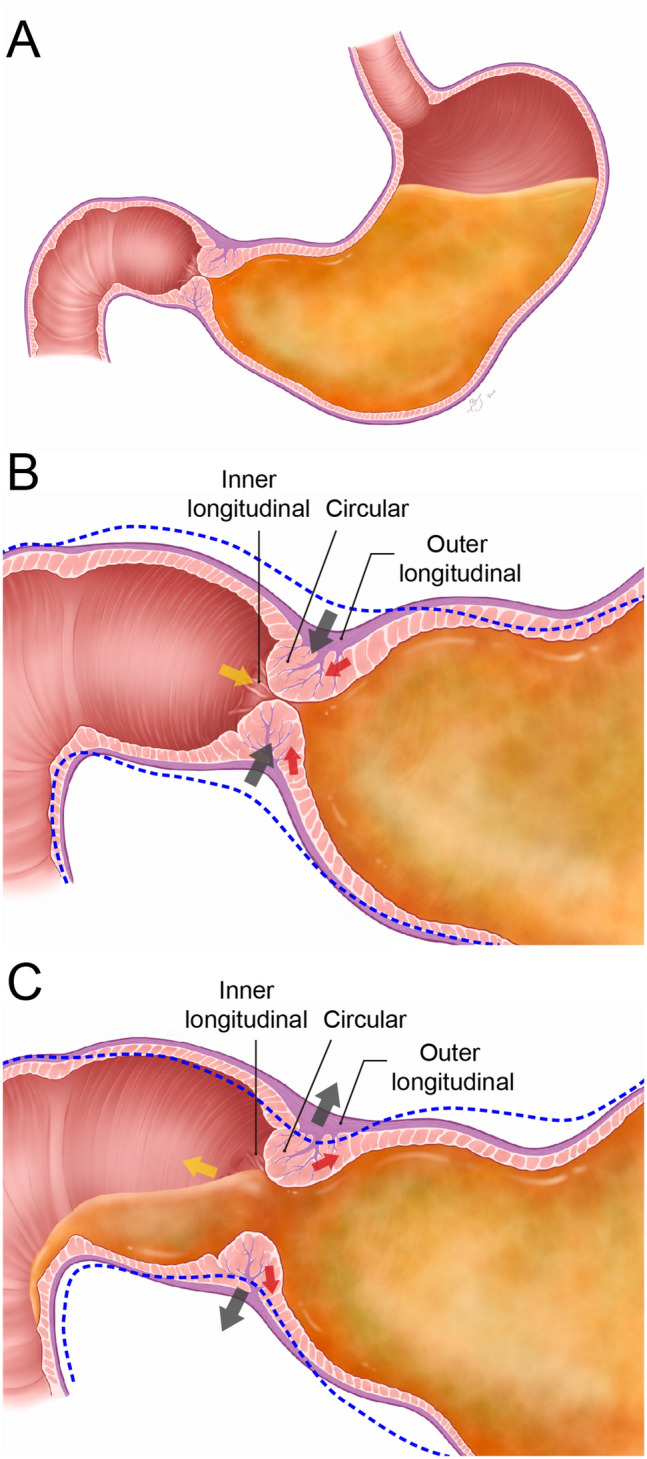
Potential steps in the contraction of the three pyloric sphincter (PS) layers. (**A**) In the early stage of the simulation, gastric contents are passing toward the PS. (**B**) As simulation continues, the three PS layers contract. Contractions of the inner (yellow arrow) and outer (red arrow) longitudinal bundles may simultaneously improve the balance between the outer and inner walls of the PS during local longitudinal shortening, enhancing PS motility and tightly closing the lumen. Contraction of the middle circular bundles (black arrow) may close the pyloric lumen. Contractions of the three PS layers may induce contraction of the oral duodenum toward the PS, combining these structures to improve length and intraluminal resistance to control the passing of gastric contents into the duodenum. (**C**) The PS then relaxes back to its original diameter and the oral duodenum moves backward, relaxing back to its original position. Blue dashed lines indicate the former PS shape and its adjacent PC and duodenum before the motility stage.

Tonic contraction of the sphincter is necessary for food digestion and the prevention of food reflux from the duodenum. While the stomach grinds food by repeated gastric peristalsis, the closed sphincter restricts the passage of food and maintains the pressure on the gastric lumen. Food entering the duodenum should be prevented from returning to the stomach. This study found a physical connection between the inner longitudinal bundles of the sphincter and the circular bundles of the duodenum. This connection can induce a synchronized contraction of the oral duodenum and the sphincter. Similarly, the connection observed on micro-CT images between the outer longitudinal bundles of the sphincter and the circular bundles of the duodenum can induce simultaneous contractions of these two bundles. These muscle connections will contract the longitudinal bundles of the sphincter and pull the duodenum, which further increases the resistance of the sphincter and canal by constricting the aboral end of the opening of the sphincter. The findings of this study support the hypothesis of a sequential contraction of all three muscle layers as the sphincter opens and closes (Fig. 9). Nevertheless, the pyloric contraction process proposed based on the present findings needs to be validated by acquiring high-resolution in vivo images from humans.

This study found a close relationship between the sphincter, antrum, and duodenum during antropyloroduodenal motility. Shafik et al. (2007)^7^ reported that sphincter distension significantly increased the antral pressure, but not the proximal stomach pressure. Weisbrodt et al. (1969)^8^ suggested that the relative contractile forces of the antrum and duodenum regulate gastric emptying to some extent. From a physiological perspective, peristaltic forces originate from the muscle bundle tone generation, which is regulated and controlled spatially and temporally by central and enteric neuromuscular interactions^9^. The connections between the longitudinal and circular bundles—inner longitudinal bundles of the sphincter and their connections with the circular bundles of the adjacent duodenum, and the longitudinal bundles of the pyloric canal and their connections with the circular bundles of the sphincter—imply an elaborate coordination between concurrent local longitudinal shortening and circular muscle contraction during peristaltic waves, which efficiently reduces the closure pressure required for antropyloroduodenal motility.

Several structures have been known to have an additional layer for improving function efficiency. For example, the ureter has an inner longitudinal and outer circular layer, but also an additional outer longitudinal muscle layer in the distal third that assists with peristalsis^10^. The vas deferens also has three muscle layers consisting of inner longitudinal, middle circular, and outer longitudinal bundles. The inner longitudinal muscle layer of the vas deferens may assist in reversing the direction of sperm movement^11,12^. The cardiac sphincter muscle in capybaras partly formed by the external longitudinal and internal oblique bundles connected to the circular layer, which structurally reinforces the pyloric ostium. This additional reinforcement of the cardiac sphincter muscle may be related with prevention of gastroesophagic food reflux during the digestive process, which requires powerful and efficient muscular control^13^. The inner longitudinal layer of the human pyloric sphincter could therefore contribute in reinforcing the pyloric motility and preventing duodenogastric reflux.

The pyloric canal’s longitudinal muscles contract first to shorten the canal, and circular muscle contraction then ejects the contents of the canal into the duodenum and backward into the stomach. Without longitudinal muscle contraction, the circular muscle functions with a minimal pumping effect. Physiologically, local longitudinal shortening focuses on circular muscle bundles, which have the highest closure pressure^5^. Local longitudinal shortening induces combined physiological and mechanical effects such as reducing the tension and power of the circular muscle bundle to as low as 10% of what would be required for peristalsis without the longitudinal muscle layer^9^. A higher degree of local longitudinal shortening would therefore imply a higher concentration of circular muscle fibers and a potentially greater closure pressure^9^, which indicates the importance of the longitudinal bundles when there is a high concentration of circular bundles.

## Conclusion

This study has revealed the complicated structure of the sphincter using microdissection, histology, and micro‐CT. The results obtained could provide new insights.

## Materials and methods

### Specimens and dissection

The stomachs were obtained from 80 embalmed Korean adult cadavers (44 males and 36 females) with a mean age at death of 69.2 years (age range, 33–95 years). The cadavers were fixed by arterial perfusion with 8% formalin.

All cadavers had been legally donated to the Catholic Kwandong University College of Medicine, and this study was conducted in accordance with the Declaration of Helsinki. No transplant donors were from a vulnerable population and all donors or their next of kin volunteered written informed consent. This study was approved by the Institutional Review Board of the Catholic Kwandong University (IRB No. CKU-20-01-0410).

Sphincters and the adjacent stomach and duodenum of 52 specimens were dissected under a surgical microscope. Sphincters and the adjacent regions were longitudinally cut through the greater curvature, lesser curvature, and both curvatures at the angle of perpendicular to the axis of lumen. The specimens were also cut between the curvatures at the angle of horizontal to the axis of lumen. The sphincters were axially cut through both curvatures at the angle of perpendicular to the axis of lumen. Mucosae and submucosae were removed from the internal sphincter surface and adjacent regions to observe muscle bundles.

### Staining for histological analysis

Histological analysis using H&E and Masson’s trichrome stains was performed on six specimens. For histological evaluation of the sphincter smooth muscle and adjacent duodenum arrangements, the specimens were stained on 5‐µm‐thick sagittal and axial sections (three of each type).

### Analysis of the sphincter using micro-CT scanning images

Another 22 specimens were consecutively immersed in 30%, 50%, and 70% ethanol solutions and then placed in a 1% phosphotungstic acid solution with 70% ethanol for 8–12 weeks. These were then scanned using micro‐CT to produce images with a pixel size of 28.64 μm^2^. These images were transferred to Mimics software (version 21.0, Materialise, Leuven, Belgium) to extract serial longitudinal and axial images for sphincter muscle arrangement analyses.

### Numerical simulations of sphincter contraction

Numerical simulations were performed to elucidate the function of the longitudinal and circular bundles of the sphincter. Flexible bundle motion can be modeled by the immersed boundary (IB) method^14^, which was originally proposed by Peskin (2002)^15^. The conventional IB method usually uses the Navier–Stokes equations to represent the structure’s motion; however, the transport equation for simple implementation and immediate reaction from the generated force was the only part used in our simulation. The details of the forcing formula were referred to in Lim and Kim (2011)^16^ and Lee et al. (2016)^17^. The following equations describe the energies and forces of the longitudinal and circular muscle motions:

$$\begin{aligned} E_{circular} \;[{\mathbf{X}}( \cdot ,\;t)] & = \frac{\sigma }{2}\int {\left( {\left| {\frac{{\partial {\mathbf{X}}}}{\partial s}} \right| - 1} \right)^{2} } ds, \\ {\mathbf{F}}_{circular} \;(s,t) & = - \frac{{\wp E[{\mathbf{X}}( \cdot ,\;t)]}}{{\wp {\mathbf{X}}(s,\;t)}}, \\ {\mathbf{F}}_{longitudinal} \;(s) & = k_{T} ({\mathbf{X}}_{T} (s) - {\mathbf{X}}(s)). \\ \end{aligned}$$

where *E*_*circular*_ is the elastic shrinking energy of the circular bundles, *F*_*circular*_ is the force accounting for variational derivatives, *F*_*longitudinal*_ is the tethered shrinking force of the longitudinal bundles, **X** is the fiber position, *s* is the parametrization, *t* is time, and σ and *k*_*T*_ are the coefficients relating to the energy and force, respectively. The connections between each longitudinal and circular bundle can be easily coupled by calculating the net force derived by the corresponding bundles using the following formula:

$${\mathbf{F}}_{connection} \;(s,\;t) = - \frac{{\wp E[{\mathbf{X}}( \cdot ,\;t)]}}{{\wp {\mathbf{X}}(s,\;t)}} + k_{T} ({\mathbf{X}}(s) - {\mathbf{X}}(s)).$$

### Ethics approval

This study was approved by the Institutional Review Board of the Catholic Kwandong University (IRB No. CKU-20-01-0410).

## Supplementary Information

Supplementary Figure 1.

Supplementary Figure 2.

Supplementary Legends.

## Data Availability

All data generated or analysed during this study are included in this published article.
