# Supplementary figures and images for: The three muscle layers in the pyloric sphincter and their possible function during antropyloroduodenal motility
Source: Sci Rep. 2021 Oct 11;11:20094. doi: 10.1038/s41598-021-99463-x (PMC8505543; doi:10.1038/s41598-021-99463-x)

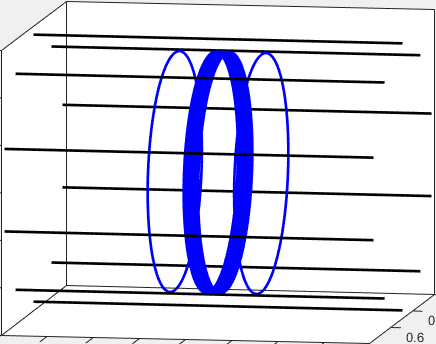

Supplement: Supplementary file 1 — Supplementary Figure 1. [file 41598_2021_99463_MOESM1_ESM.gif]

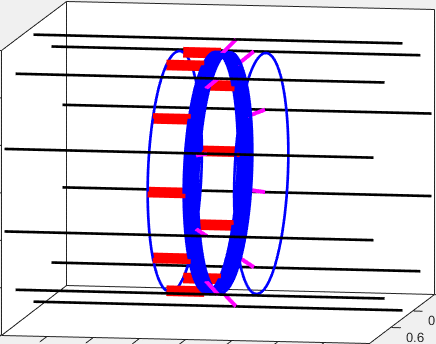

Supplement: Supplementary file 2 — Supplementary Figure 2. [file 41598_2021_99463_MOESM2_ESM.gif]
