# Supplementary material for: The three muscle layers in the pyloric sphincter and their possible function during antropyloroduodenal motility
Source: Sci Rep. 2021 Oct 11;11:20094. doi: 10.1038/s41598-021-99463-x (PMC8505543; doi:10.1038/s41598-021-99463-x)
Supplement: Supplementary file 3 — Supplementary Legends. [file 41598_2021_99463_MOESM3_ESM.docx]

**Supplementary Figure 1.**

(**A**) A simulation of PS muscle fiber (thicker blue) contractions with the inner layer of longitudinal fiber (red) and the middle layer of longitudinal fibers (pink) is presented in an online video. Left blue, Duodenum; Right blue, pyloric canal.

(**B**) A simulation of PS muscle fiber (thicker blue) contractions without the inner layer of longitudinal fiber and the middle layer of longitudinal fibers is presented in an online video. Left blue, Duodenum; Right blue, pyloric canal.
